# Intracellular radar: Understanding γδ T cell immune surveillance and implications for clinical strategies in oncology

**DOI:** 10.3389/fonc.2022.1011081

**Published:** 2022-09-23

**Authors:** Anne B. C. Cherry, Nicholas A. Gherardin, Hafiz I. Sikder

**Affiliations:** ^1^ Axiom Healthcare Strategies, Princeton, NJ, United States; ^2^ Department of Microbiology and Immunology, Peter Doherty Institute for Infection and Immunity, University of Melbourne, Melbourne, VIC, Australia

**Keywords:** γδT, immunotherapy, anticancer, intracellular, surveillance, clinical, chimeric antigen receptor, gamma delta (γδ) T cells

## Abstract

T cells play a key role in anticancer immunity, with responses mediated through a diversity of αβ or γδ T cell receptors. Although αβ and γδ T cells stem from common thymic precursors, the development and subsequent biological roles of these two subsets differ considerably. γδ T cells are an unconventional T cell subset, uniquely poised between the adaptive and innate immune systems, that possess the ability to recognize intracellular disturbances and non-peptide-based antigens to eliminate tumors. These distinctive features of γδ T cells have led to recent interest in developing γδ-inspired therapies for treating cancer patients. In this minireview, we explore the biology of γδ T cells, including how the γδ T cell immune surveillance system can detect intracellular disturbances, and propose a framework to understand the γδ T cell-inspired therapeutic strategies entering the clinic today.

## Introduction

The field of cancer immunology has grown around the idea that cancer elimination is a key role of the human immune system (1). Recent discoveries have revealed that T cells play a central role in immune surveillance, with responses mediated through a diversity of αβ or γδ T cell receptors (TCRs) (2). Despite sharing common thymic precursors, the development and subsequent biological roles of αβ and γδ T cells differ considerably (2). While αβ T cell activation typically induces adaptive and long-lasting responses, γδ T cells can respond rapidly and kill tumor cells directly (2, 3). In order to evade the host immune response, tumors have evolved diverse escape mechanisms, such as overexpression of programmed death ligand-1 (PD-L1) or immune exclusion, which are employed following malignant transformation (4–7). This convergent evolution of tumors toward a few immune escape mechanisms across a wide variety of cancer types underscores the central role of immune surveillance in preventing tumor growth, and has enabled the broad efficacy of immune checkpoint inhibitors (8).

Disturbances in intracellular processes, including metabolism, lipid composition and processing, nucleic acid packaging, glycans, biomechanical properties, and endoplasmic reticulum stress are key characteristics of tumor cells (8–13). Non-protein cellular signals of cancer have been recognized for over a century, since the metabolic shift from mitochondrial respiration to aerobic glycolysis was identified in the 1920s by Otto Warburg and colleagues as a fundamental trait of cancer (14). While “conventional” αβ T cells detect tumor-derived peptide antigens to identify malignant cells, a heterogeneous family of “unconventional” T cells recognizes non-peptide-based antigens to facilitate tumor recognition (15).

γδ T cells are one such unconventional T cell subset, uniquely poised to have both adaptive and innate immune functions (16). Like αβ T cells, γδ T cells express a TCR on their cell surface that associates with the CD3 complex (16). During thymic development, successful rearrangement of a γδ TCR drives commitment to the γδ lineage *via* a different pathway than that of αβ T cells, resulting in the innate-like phenotypic features that differentiate γδ T cells from their αβ counterparts (3). Despite the discovery of γδ T cells over three decades ago, the mechanisms by which γδ T cells recognize antigens remain poorly understood (17). Nonetheless, an emerging paradigm suggests that γδ TCRs recognize and respond to modulation in cell surface “sentry” molecules indicative of intracellular stress (7, 18). The unique features of γδ T cells have led to widespread interest in exploring their potential application in the development of novel therapeutics for cancer (3).

In this minireview we summarize the current understanding of how the human γδ T cell immune surveillance system can detect intracellular disturbances and explore new therapeutic approaches undergoing preclinical and clinical development.

### The science of γδ T cells: Intracellular radar

Despite decades of research, our understanding of the mechanisms by which the immune system detects intracellular disturbances is still incomplete (17). However, recent research suggests that γδ T cells monitor intracellular health *via* detection of transmembrane sentry proteins (18). When a cell’s sentry proteins indicate intracellular stress, γδ T cells can promptly deliver a cytotoxic response to eliminate the stressed cell, as well as release cytokines to orchestrate a broader immune response (3, 17).

**Figure 1:**
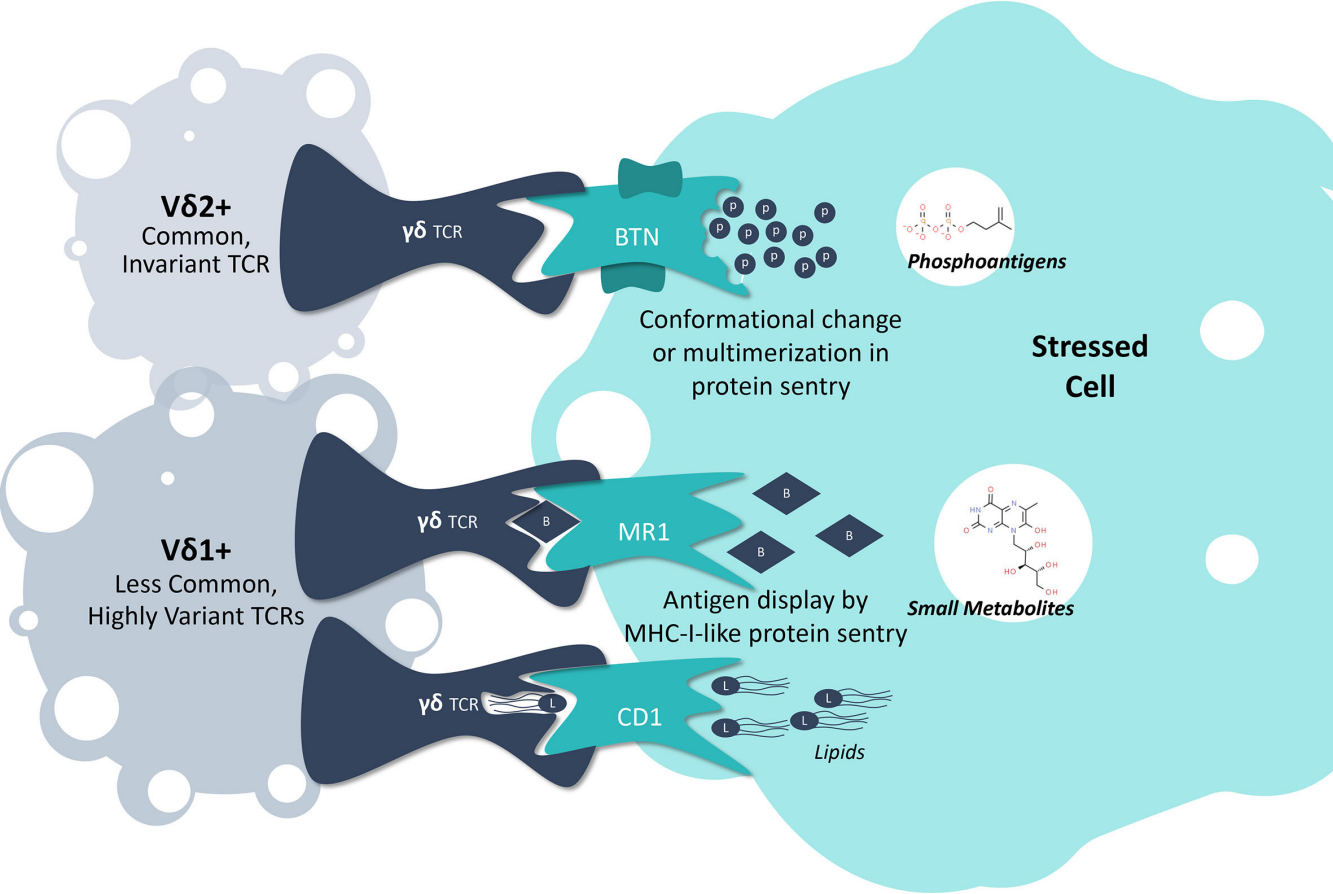
γδ T Cell Detection of Intracellular Stress. A collection of sentry proteins monitor the intracellular space for specific molecular signs of stress, including butyrophilins monitoring for phosphoantigen levels, MR1 monitoring for small metabolites indicative of tumor development or cancer, and CD1 monitoring for lipids. By interacting with these protein sentries, γδ T cells can detect when a cell is under stress and initiate an effector response.

Human γδ T cells are themselves heterogeneous, and are broadly categorized into two major subsets based on their distinct antigen-recognition and phenotypic features, described according to the antigen-recognition domain of their TCR-δ chains: Vδ2+ and non-Vδ2 (19, 20). The latter consists primarily of Vδ1+ cells, although other less well-characterized cells expressing Vδ3-Vδ8 also exist (20, 21). T cells carrying the Vδ2+ chain are more common in circulation, and respond to intracellular accumulation of tumor-associated metabolic byproducts by interacting with a family of sentry proteins called butyrophilins (BTNs) (22, 23). Non-Vδ2 cells, on the other hand, respond to a variety of stress-associated sentry proteins, the most well-characterized of which are major histocompatibility complex (MHC) class I-like proteins (19, 20, 24). Each γδ T cell subset also possesses unique tissue localization and homing capabilities, as evidenced by Vδ2+ cells tending to be more enriched in blood, versus non-Vδ2 cells, which are often found enriched in tissues such as the gut (25, 26). Here, we provide an overview of these two distinct groups of γδ T cells, including what is known about intracellular stress responses and how γδ TCRs are able to detect these changes (**Figure 1**).

### Phosphoantigens, butyrophilins, and Vδ2+ γδ T cells

Most Vδ2+ γδ T cells express an invariant TCR, imbuing responsiveness to the intracellular presence of low molecular weight, phosphorylated adducts or “phosphoantigens” (pAgs) (27). pAgs are found in healthy cells at low levels as part of the mevalonate synthesis pathway, but elevated levels signal cellular dysregulation (28). For example, highly potent pAgs such as (E)-4-Hydroxy-3-methyl-2-but-2-enyl pyrophosphate are generated as byproducts of microbial isoprenoid synthesis *via* the methylerythritol phosphate pathway, and thus represent a molecular signature of microbial infection (29). Vδ2+ γδ T cells also respond to mammalian pAgs of lower potency, such as the isoprenoid precursor isopentyl pyrophosphate, that accumulate from an overactive mevalonate metabolic pathway during cancer development (30). Accordingly, Vδ2+ γδ T cells act as immune sentinels, monitoring intracellular stress *via* a conserved molecular pattern.

The precise mechanism by which pAgs are recognized by the γδ TCR has been a conundrum for the field. Recent studies, however, have revealed that the process is dependent on two members of the BTN protein family, BTN3A1 and BTN2A1 (31, 32). Here, pAgs bind to the intracellular domain of BTN3A1, resulting in an undefined process of extracellular remodeling to reveal an antigenic target for the TCR, which includes direct interactions with BTN2A1 (25, 31, 32). This process is a distinct mode of antigen-sensing compared to αβ TCR recognition of peptide-MHC, although the molecular details are yet to be revealed (27).

### Other intracellular stressors, sentry proteins, and non-Vδ2 γδ T cells

Like Vδ2+ cells, non-Vδ2 γδ T cells exhibit both antimicrobial and antitumor potential at a population level; however, the antigens recognized by non-Vδ2 cells are less well understood (19, 20). Unlike their invariant Vδ2+ counterparts, non-Vδ2 γδ T cells exhibit extensive TCR diversity *via* TCR-γ chain pairing and more extensive complementarity-determining region 3 variation, and thus have highly variable antigen-recognition potential (19, 20). Despite a paucity of literature on non-Vδ2 antigen identification, the antigens described to date appear to be an array of molecules indicative of various forms of intracellular stress (18).

One major group of antigens consists of monomorphic MHC I-like molecules, including the CD1 family and the MHC-related protein 1 (MR1) (24, 33–38). CD1 sentry proteins are involved in the presentation of lipid antigens to various subsets of Vδ1+ γδ T cells (33–38). MR1 proteins present microbial vitamin-B metabolite antigens, as well as undefined tumor-associated antigens to various subsets of non-Vδ2 γδ T cells (24, 39–42). While research on CD1/MR1-γδ T cell interactions continues, early evidence indicates that subsets of γδ T cells also respond to a plethora of other intracellular stress signals through sentry binding partners, such as the endothelial cell protein C receptor (EPCR), MHC class I chain-related protein A, and UL16-binding protein, which are MHC I-like, and annexin A2, EphA2, and phycoerythrin, most of which still remain poorly understood (43–48). Taken together, it appears that non-Vδ2 γδ T cells interact with a wide range of sentry proteins to detect a broad array of intracellular stresses.

The mode of γδ TCR recognition of MHC I-like molecules appears more varied than αβ TCRs, which conform to a relatively rigid docking mode atop peptide-MHC complexes (49). In contrast, γδ TCRs appear to dock across the entire antigen-presenting platform, and in some cases, adopt highly unusual modes of recognition (41, 43). For example, some MR1-restricted TCRs dock with MR1 underneath the antigen-binding cleft, while others such as EPCR-restricted clones are able to recognize the underside of the antigen-binding platform (41, 43). Thus, while these sentries are bona fide antigen-presenting molecules, and many γδ TCRs exhibit antigen dependence, other TCRs seem to bind in an antigen-independent manner (50).

### Role in anticancer immunosurveillance

The research described here has shed light on how sentry proteins monitor for intracellular stress and convey that information to γδ T cells. While it remains an open question whether certain parts of these systems respond preferentially to infection, cancer, or other stressors, there is abundant evidence demonstrating that γδ T cells play an active role in cancer surveillance. This includes observation of γδ T cell enrichment across a broad range of tumor types, *in vitro* evidence of direct Vδ2+ T cell tumor cell killing in a BTN-dependent manner, and an analysis of gene expression signatures from 18,000 tumors that demonstrated the γδ T cell signature as the most favorable for patient survival among 22 immune cell subtypes (51). γδ T cell tumor recognition and elimination is also supported by the expression of receptors associated with natural killer cell activity, including NKG2D, DNAM-1, and other natural cytotoxicity receptors such as NKp30, NKp44, and NKp46.

While the evidence is clear that γδ T cells play an active role against tumors, an important unanswered question is how tumors evade γδ T cell recognition and destruction. More broadly, identifying the mechanisms by which tumors evade immunological surveillance is a rapidly evolving area of research, and disentangling the separate contributions of the αβ vs. γδ T cell surveillance systems remains difficult (52). There are a number of mechanisms by which tumor cells directly or indirectly suppress T cell immune responses, many of which likely apply to both the αβ and γδ T cell compartments (53). In particular, expression of immune checkpoints such as PD-L1 have been successfully targeted with cancer immunotherapies (8). Additionally, cancer immunoediting of surface-expressed molecules involved in antigen presentation to γδ T cells has been observed, which could represent another mechanism by which tumors evade recognition by γδ T cells (54–56). Finally, in some environments, γδ T cells could also play an immunosuppressive role, which may contribute to tumor immune evasion (57).

Overall, these advances show that γδ T cells monitor the intracellular well-being of tissues *via* a network of sentry proteins. When a cell experiences intracellular distress, γδ T cells can directly kill the cell and/or recruit other arms of the immune system to respond. While γδ T cells are involved in routine anticancer immunosurveillance, ongoing research is focused on improving our understanding of the underlying mechanisms.

## Discussion: Clinical strategies and progress to date

There is strong biological rationale for harnessing the γδ T cell surveillance system to enhance antitumor immunity. Despite unprecedented clinical success with checkpoint inhibitor therapies over the past decade, many patients do not respond and most will eventually relapse (58). While other αβ T cell-based approaches are being developed to address these limitations, such as engineered chimeric antigen receptor (CAR)-T cells, there is increasing interest among investigators to develop novel γδ T cell-based therapeutics with superior activity to fight against human cancers (7). However, it is yet unknown how γδ T cell-inspired therapies will compare to other existing and emerging types of cell therapies.

**Figure 2:**
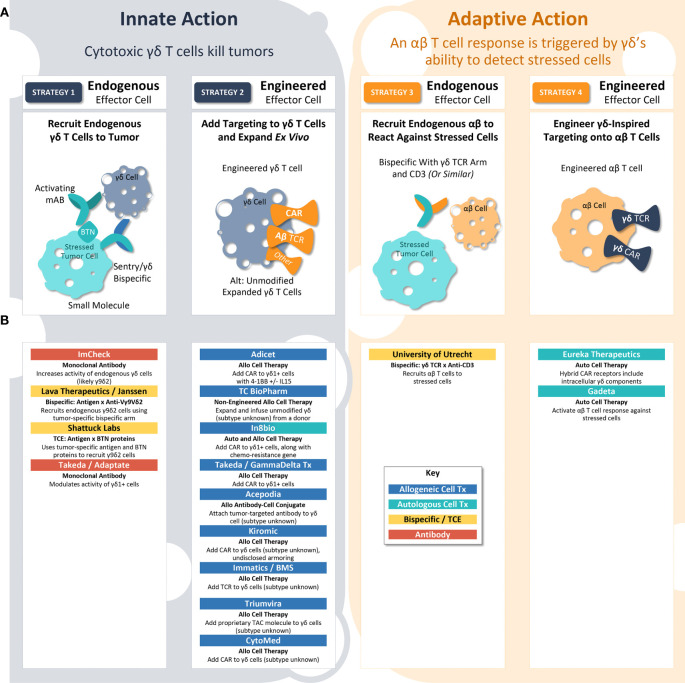
γδ T Cell-Inspired Clinical Strategies. **(A)** γδ T cell biology may be employed to eliminate tumors by four different strategies. In strategy 1, endogenous γδ T cells kill the tumor. In strategy 2, exogenous engineered γδ T cell therapies kill the tumor. In strategy 3, γδ T cell receptor biology is used to recruit an endogenous αβ T cell adaptive response to stressed tumor cells. In strategy 4, γδ T cell receptor biology is used as a targeting strategy for traditional αβ CAR-T cells to identify and attack stressed tumor cells. In each of these strategies, researchers can either employ validated protein constructs (e.g., CARs using GP3C – 4-1BB – CD3ζ from Adicet) or engineer innovative protein structures (e.g., fusion protein chains of CD19 – Fc – BTN3A1/BTN2A1 from Shattuck Labs) to maximize anti-tumor activity. **(B)** Researchers and companies leading investigation into each clinical strategy.

A broad interventional toolkit is investigating the antitumor activity of γδ T cell surveillance, including cell therapy, protein engineering, and direct modulation by monoclonal antibody (mAb) or small molecules (**Figure 2**). At present, there is no aligned vision for how γδ T cell biology can best be leveraged to fight cancer, and thus researchers are manipulating various aspects of the immunological surveillance system in an effort to maximize antitumor activity (59). In the following section, we propose a framework for understanding the γδ T cell-inspired therapeutic strategies in clinics today.

### Innate action: γδ T cells kill tumor cells (strategies 1 & 2)

γδ T cells can directly kill tumor cells with innate-like cytotoxicity. In this way, therapeutic approaches leveraging the ability of γδ T cells to kill tumors directly are similar to CAR-NK approaches, which direct natural killer cells’ innate cytotoxicity against cancer (60). Innate action therapies seek to either recruit endogenous γδ T cells to the tumor (Strategy 1) or expand and engineer γδ T cells *ex vivo* to deliver as therapy (Strategy 2).

#### Strategy 1: Recruit endogenous γδ T cells

A strategy involving endogenous γδ T cells is complicated by the fact that the patient’s immune system has already allowed the tumor to grow. To overcome this challenge, researchers are working on ways to reinvigorate the endogenous γδ T cell antitumor response. For example, researchers at ImCheck are attempting to increase signaling from the sentry protein using a mAb activating BTN3A (61). Other examples include Lava Therapeutics and Shattuck Labs, both of which are developing bispecific proteins designed to bring γδ T cells within close proximity to tumors (62). Two divergent strategies are being employed to attract the γδ T cells (1): Lava Therapeutics has developed an anti-Vγ9Vδ2 nanobody arm; and (2) Shattuck Labs is utilizing a piece of Vγ9Vδ2’s natural ligand, BTN, as part of its heterodimer (62, 63). If these approaches prove successful, they will have the distinct advantage of being off-the-shelf, protein-based therapeutics, which are easier to manufacture and deliver than cell therapies. Of note, these examples rely on Vδ2+ cells, rather than Vδ1+ cells, and thus assuming that BTN-based signaling will provide maximal antitumor efficacy, but not considering other types of intracellular stress.

#### Strategy 2: Expand and engineer γδ T cells *ex vivo* to deliver as therapy

Another innate strategy involves adding a CAR or TCR to the surface of a γδ T cell body for increased specificity against cancer cells. One advantage of this approach is that CARs or TCRs that have been clinically validated as cell therapies can be brought into the γδ T cell paradigm. For example, Adicet is collecting and expanding Vδ1+ T cells from donors, then adding a CAR construct targeted against CD20 with a 4-1BB costimulatory domain to these Vδ1+ cells (64). Another company, In8bio, is seeking to harness γδ T cells’ natural ability to kill stressed cells by including a chemoresistance gene in its cell therapy, enabling chemotherapy to be administered without damaging the γδ-based CAR-T therapy (65). Most companies employing Strategy 2 are developing allogeneic cell therapies, leveraging γδ T cells’ natural avoidance of graft vs. host disease, which can generate hundreds or thousands of doses in a single production run. However, the long-term success of these approaches rests on a crucial question: Will cell therapies using γδ T cells demonstrate the same *in vivo* expansion, persistence, and long-term activity that other CAR-T therapies offer? Currently, this question remains unanswered. The success of many CAR-T products available today is attributable to the adaptive immune behavior of the carrier αβ T cell, and whether the more innate-like γδ T cell will deliver the same results has yet to be seen.

### Adaptive action: αβ T cells kill tumor cells (strategies 3 & 4)

Instead of using γδ T cells directly, some investigators are seeking to exploit their ability to detect and respond to intracellular stress. As a field, cell therapy has remained highly dependent on tumor cell-specific antigens; however, with γδ TCR biology, investigators are seeking to link the innate and adaptive immune systems, using sentries’ stress signals as an “antigen” to trigger adaptive immune activity. These adaptive action therapies can rely on either activation of endogenous αβ T cell responses against stressed cells (Strategy 3) or an αβ CAR-T therapy built using a γδ-inspired CAR or TCR (Strategy 4).

#### Strategy 3: Activation of endogenous αβ T cells against stressed cells

CD3 bispecific proteins exert antitumor effects by binding to a cancer-specific antigen at the same time they bind to CD3, a molecule expressed on both CD8+ and CD4+ T cells. This binding event allows the T cells to directly kill the cancer cell, overcoming usual antigen presentation and MHC matching processes. Researchers from the University of Utrecht have developed a bispecific protein that includes both a CD3-binding arm and Vγ9Vδ2 TCR (66). As previously discussed, Vδ2+ TCRs bind to activated BTN2A1 protein sentries, bringing CD8+ and CD4+ T cells within close proximity to stressed tumor cells. Akin to Strategy 1, this approach relies entirely on the Vγ9Vδ2/BTN axis, rather than including Vδ1+ cells, and if successful it could offer logistical and manufacturing advantages.

#### Strategy 4: αβ CAR-T therapy built using a γδ-inspired CAR or TCR

The most nascent strategy in development is the addition of γδ TCRs to αβ T cells. In theory, this approach could merge the powerful, long-lasting benefit of traditional CAR-T therapy with γδ T cells’ ability to detect intracellular stress in a non-MHC-restricted manner. However, investigators must address at least three key challenges before this strategy can become a reality: 1) Identify a γδ TCR sequence that can reliably recognize a stressed sentry protein, whether Vδ2+ or Vδ1+. Sequencing of tumor-reactive γδ TCRs is still in early stages, and it is not yet known whether a single TCR would be broadly effective or if different tumors would require different TCRs. The specificity of the TCR for stressed vs. non-stressed cells will also be crucial, as this will provide a benchmark for the degree of off-target activity. Early work in this area was conducted by Wei He and colleagues, who added tumor-specific γδ TCRs to αβ T cells and observed a reduction in tumor growth in a mouse model (67). 2) Determine which parts of the chimeric receptor should be derived from γδ T cells vs. traditional CAR/TCR molecules, and how these components impact the therapy’s behavior *in vivo*. For example, Gadeta is developing αβ T cells equipped with γδ TCRs to detect stress signals from sentry proteins (68). However, Eureka Therapeutics is using a hybrid CAR receptor that includes γδ TCR sequence on the intracellular portion of the CAR, while the extracellular portion is a traditional anti-CD19 (69). The impact of receptor designs on safety, including cytokine release syndrome, should also be determined. 3) Finally, αβ-based therapies will initially be limited to the autologous setting, until an extra step is introduced to genetically modify the source material. Accordingly, both γδ-inspired αβ CAR-T cells in the clinic today are autologous.

## Conclusion and remaining questions

γδ T cell-based therapies represent an emerging class of anticancer treatments, with a growing pipeline of investigational agents in varied stages of clinical development. In this minireview, we explored the biology of γδ cells and have proposed a framework for understanding the γδ T cell-inspired therapeutic strategies currently in use. Despite recent progress, there remains a need for more comprehensive understanding of γδ immune surveillance, as the success of clinical strategies depends on several key questions that remain unanswered. These questions include: What protein sequence will be the most effective bait to recruit endogenous γδ T cells? Will cell therapies using γδ T cell biology demonstrate the same expansion and long-term activity that traditional CAR-T therapies have shown? Is there a γδ TCR sequence that would consistently recognize stressed cells across patients, and if so, how can it be identified? Can γδ T cell biology be used to design a traditional CAR-T therapy that activates the adaptive immune system against stressed cells? The answers to these questions will determine which γδ T cell-inspired clinical strategy will be more effective in the fight against cancer.

## Author contributions

AC and NG drafted the manuscript and designed the figures. HS discussed and reviewed the final manuscript. All authors contributed to the article and approved the submitted version.

## Funding

The authors received no financial support for the research or authorship of this article. NAG was supported by an Australian Research Council Discovery Early Career Award Fellowship (DE210100705). Publication was supported by Axiom Healthcare Strategies, LLS (Princeton, NJ).

## Acknowledgments

Medical writing and editorial support was provided by Caleb Rans, of Rans Medical Communications (Vancouver, Canada) and was funded by Axiom Healthcare Strategies.

## Conflict of interest

The authors declare that the research was conducted in the absence of any commercial or financial relationships that could be construed as a potential conflict of interest.

## Publisher’s note

All claims expressed in this article are solely those of the authors and do not necessarily represent those of their affiliated organizations, or those of the publisher, the editors and the reviewers. Any product that may be evaluated in this article, or claim that may be made by its manufacturer, is not guaranteed or endorsed by the publisher.
